# Essential functions of iron-requiring proteins in DNA replication, repair and cell cycle control

**DOI:** 10.1007/s13238-014-0083-7

**Published:** 2014-07-08

**Authors:** Caiguo Zhang

**Affiliations:** Department of Biochemistry and Molecular Genetics, University of Colorado School of Medicine, Aurora, CO 80045 USA

**Keywords:** iron-requiring protein, DNA replication, DNA repair, cell cycle, iron homeostasis

## Abstract

Eukaryotic cells contain numerous iron-requiring proteins such as iron-sulfur (Fe-S) cluster proteins, hemoproteins and ribonucleotide reductases (RNRs). These proteins utilize iron as a cofactor and perform key roles in DNA replication, DNA repair, metabolic catalysis, iron regulation and cell cycle progression. Disruption of iron homeostasis always impairs the functions of these iron-requiring proteins and is genetically associated with diseases characterized by DNA repair defects in mammals. Organisms have evolved multi-layered mechanisms to regulate iron balance to ensure genome stability and cell development. This review briefly provides current perspectives on iron homeostasis in yeast and mammals, and mainly summarizes the most recent understandings on iron-requiring protein functions involved in DNA stability maintenance and cell cycle control.

## Introduction

In most eukaryotic cells, iron is necessary to facilitate the assembly of functional Fe-S cluster proteins, heme-binding proteins, and ribonucleotide reductases (RNRs) (Dlouhy and Outten, 2013; Heath et al., 2013). These iron-requiring proteins are abundantly present in mitochondria, cytosol, and nucleus; such proteins diversely function in electron transfer, ribosome maturation, DNA replication and repair, and cell cycle control (Kaplan et al., 2006; Ye and Rouault, 2010; White and Dillingham, 2012).

Unbalanced iron levels always affect the physiology of organisms. For instance, excess intracellular iron may result in the generation of reactive oxygen species (ROS), which can damage lipids, proteins, DNA; this adverse effect may eventually lead to genome instability and cell death in almost all organisms (Orrenius et al., 2011; Romero et al., 2014; Turrens, 2003). On the other hand, iron deficiency is extremely common in different species. Iron deficiency caused anemia is one of the major public health problems, particularly in children and pregnant women (Denic and Agarwal, 2007; Miller, 2013). In plants, the photosynthesis process is highly dependent on iron. Iron deficiency often reduces the amount of electron-transferring complexes, increases proteins involved in carbon fixation, and causes chlorosis (Lopez-Millan et al., 2013; Solti et al., 2008). In budding yeast *Saccharomyces cerevisiae*, iron deficiency leads to the dysfunction of iron-dependent enzymes, hemoproteins and Fe-S proteins, thereby altering glucose metabolism and biosynthesis of amino acid and lipid (Philpott et al., 2012; Shakoury-Elizeh et al., 2010).

## Iron homeostasis in yeast and mammals

In eukaryotes, cellular iron homeostasis is achieved via strictly controlled systems for iron uptake at the plasma membrane and for eliciting balanced iron distributions among cellular compartments. In addition, the mammals should maintain systemic iron homeostasis by coordinately regulating iron absorption, storage and recycling, except keeping cellular iron balance.

### Cellular iron homeostasis in yeast

**Figure 1:**
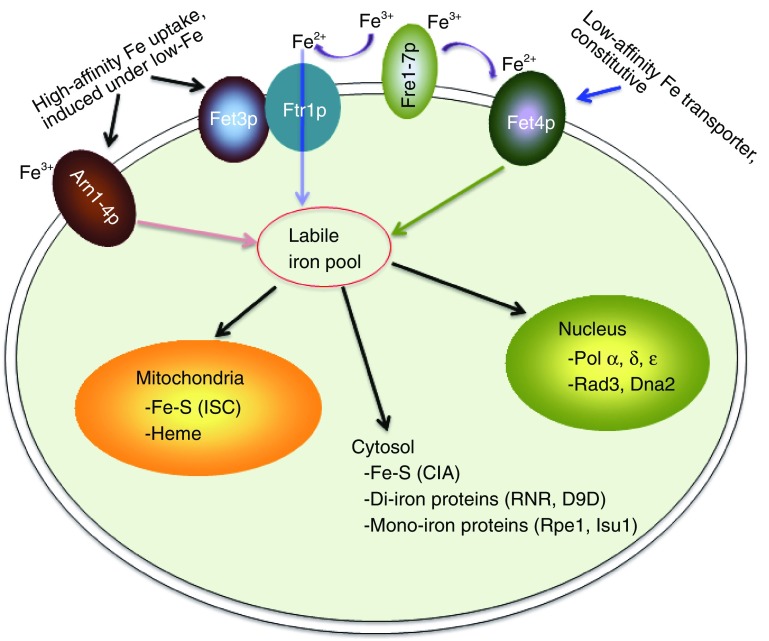
**Iron uptake and utilization inside the cell**. Yeast cells obtain iron through low-affinity (Fe-repleted condition, Fet4p) and high-affinity systems (low Fe condition, Fet3p/Ftr1p and Arn1-4 proteins). Both Fet4p and Fet3p/Ftr1p can only transport Fe^2+^, and these processes require the prior reduction of Fe^3+^ to Fe^2+^ by surface reductases (Fre1 to Fre7) (Herbik et al., 2002; Holmes-Hampton et al., 2013; Wu et al., 2005; Yun et al., 2001). The cytosolic “labile iron pool” is utilized by Fe-S proteins, hemoproteins, ribonucleotide reductases (RNRs), and other iron-requiring proteins that localize in different cellular compartments

Yeast cells acquire iron at the plasma membrane by utilizing high- and low-affinity iron uptake systems (Fig. 1). Under iron sufficient conditions, yeast cells mainly obtain iron through the low-affinity plasma membrane transporter Fet4. This process involves surface reductases (Fre1-Fre7), which can reduce Fe^3+^ to Fe^2+^ (Herbik et al., 2002; Holmes-Hampton et al., 2013; Wu et al., 2005; Yun et al., 2001). Under low iron conditions, yeast cells obtain iron via two independent high-affinity systems, particularly Fet3/Ftr1 complex and Arn1-4 proteins (Philpott, 2006; Rutherford et al., 2001; Yun et al., 2000a, b). The Fet3/Ftr1 proteins only deliver Fe^2+^ and their transcriptions are controlled by the Fe-responsive transcription factor Aft1 (Dlouhy and Outten, 2013; Hamza and Baetz, 2012). The Arn1-4 protein-dependent system becomes active in the absence of Fet3 protein, and this system is responsible for ferric siderophore uptake (Emerson et al., 2002; Heymann et al., 2000; Lesuisse et al., 1998; Yun et al., 2000a, b). When iron enters into the cytosol, it is present in a bioavailable form, namely, “labile iron pool” (LIP) (Fig. 1) (Muhlenhoff et al., 2010).

**Figure 2:**
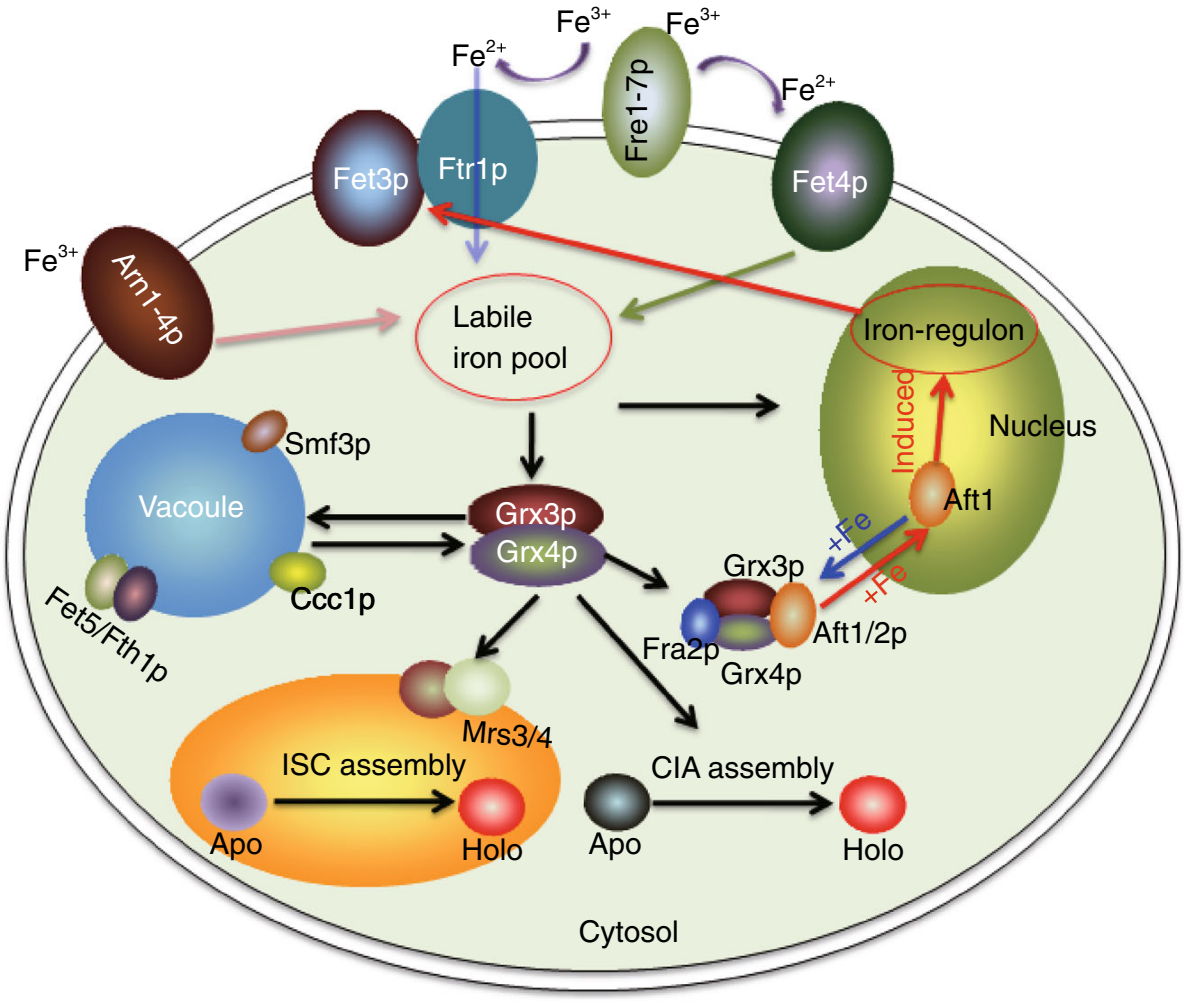
**Iron uptake, intracellular trafficking and regulation in** ***S. cerevisiae***. Iron uptake is performed at the plasma membrane by iron transporters. When iron enters into cytosol, it exists in Fe-S clusters in “labile iron pool” (Muhlenhoff et al., 2010), which is subsequently donated to cytosolic iron-dependent apoproteins through monothiol Grx3p/4p to form holoproteins. Meanwhile, the mitochondrial iron transporters Mrs3p/4p and the vacuolar iron transporter Smf3p, Ccc1p and Fet5p/Fth1p can also accept iron from Grx3p/4p. Grx3p/4p can interact with Fra2p and Aft1p/Aft2p, and the Grx3p/4p- bound Fe-S clusters may function as sensors for the cytosolic iron pool (Lill and Mühlenhoff, 2008; Muhlenhoff et al., 2010). In low-Fe condition, Aft1p can shuttle between the cytosol and the nucleus in an iron-responsive manner, and functions as a transcriptional activator of iron regulon genes, which subsequently activate high-affinity iron uptake systems (Berthelet et al., 2010; Lill et al., 2012)

Intracellular free iron is mainly stored in the vacuole, where it can be dynamically imported and exported by high- and low-affinity transporters (Fig. 2). The high-affinity vacuolar iron transport complex Fet5/Fth1 is a homologue of Fet3/Ftr1, and specifically responds to low iron (Amillet et al., 1996; Kaplan et al., 2006; Li and Kaplan, 1998; Li et al., 2008; Urbanowski and Piper, 1999). By contrast, the low-affinity vacuolar iron transporter Smf3 (DMT1 in mammals), which is a homologue of the cell membrane transporter Smf1, mainly functions in iron rich conditions (Portnoy et al., 2000; Portnoy et al., 2002). Interestingly, the Fet5/Fth1 complex and Smf3 are transcriptionally regulated by *AFT1* and *AFT2* (Li et al., 2008). Under Fe-deficient conditions, *AFT1* and *AFT2* activate the expressions of vacuolar iron exporter genes, particularly *FET5/FTH1* and *SMF3*; as a result, cytosol iron is increased and vacuolar iron is decreased (Dlouhy and Outten, 2013). Moreover, iron storage in yeast requires vacuolar iron transporter Ccc1 and its expression is correlated with low iron. Activated *AFT1* and *AFT2* genes can induce *CTH2*, which subsequently binds to the 3′-untranslated region (UTR) of *CCC1* and destabilizes its corresponding mRNA; the expression of *CCC1* is then decreased (Li et al., 2001; Martinez-Pastor et al., 2013; Philpott et al., 2012). In addition, Yap5 has been indicated to function as an iron-responsive transcriptional activator that regulates vacuolar iron storage (Li et al., 2008).

Aft1 localizes in the cytosol under iron rich conditions, whereas it accumulates in the nucleus when iron is low (Yamaguchi-Iwai et al., 2002; Ueta et al., 2012; Fig. 2). This nucleus-to-cytoplasm shuttling process is highly dependent on cytosolic monothiol glutaredoxins (Grx3 and Grx4), and the BolA-like protein Fra2 (Li et al., 2009). Grx3 (PICOT in mammals) and Grx4 are required to provide bioavailable iron for the assembly of many iron-containing holoproteins such as hemoproteins, Fe-S proteins, and RNR (Haunhorst et al., 2013; Mühlenhoff et al., 2010; Zhang et al., 2014a, b). Importantly, Grx3/4 can specifically interact with the CDC motifs in Aft1/2, and also can bind to Fra2 to form [2Fe-2S]-bridged heterodimers (Li et al., 2009; Ueta et al., 2007). The Fra2-Grx3/4 complex is possibly implicated in Aft1/2 sensing of the cellular iron status (Dlouhy and Outten, 2013). Under iron rich conditions, the Fra2-Grx3/4 complex binds to a Fe-S cluster, causing Aft1/2 oligomerization. Aft1 consequently shuttles to the cytosol through Msn5 and deactivates iron regulon. Under low iron conditions, the Fra2-Grx3/4 complex cannot inhibit Aft1/2 activities. Moreover, Aft1 moves to the nucleus and activates the iron regulon to increase cellular iron levels (Fig. 2) (Li et al., 2012; Lill et al., 2012; Poora et al., 2014).

### Systemic and cellular iron homeostasis in mammals

Iron homeostasis in mammals includes systemic and cellular regulations, which have been recently summarized in detail (Andrews and Schmidt, 2007; Pantopoulos et al., 2012). Systemic iron regulatory processes occur in the following several steps: (1) iron absorption starts at duodenal enterocytes and functions in macrophage iron recycling and hepatocyte iron mobilization (Beaumont, 2010; Zhang, 2010); (2) intestinal cells then absorb iron via divalent metal transporter 1 (DMT1), which requires prior reduction of Fe^3+^ to Fe^2+^ by duodenal cytochrome b (DcytB) (Sharp and Srai, 2007; Pantopoulos et al., 2012); (3) spleenic reticuloendothelial macrophages control iron recycling from senescent red blood cells (Soe-Lin et al., 2009; Pantopoulos et al., 2012); (4) plasma transferrin (Tf) absorbs and circulates iron in the body (Pantopoulos et al., 2012); and (5) hepatic hormone hepcidin controls iron efflux by regulating the stability of ferroportin (Pantopoulos et al., 2012).

For cellular iron homeostasis, most mammalian cells acquire iron via Tf to form holo-Tf (Anderson and Vulpe, 2009; Dunn et al., 2007), which further binds to transferrin receptor-1 (TfR1) on the iron-consuming cell membrane. The holo-Tf-TfR1 complex is then internalized by receptor-mediated endocytosis (Lill et al., 2012) and acidified in the endosome. As a result, the release of Fe^3+^ from holo-Tf is facilitated (Zhao et al., 2010). The Fe^3+^ should be reduced to Fe^2+^ by a six-transmembrane epithelial antigen of the prostate 3 (Steap3) before this form of iron can be transported into the cytoplasm by DMT1 or transient receptor potential protein (TRPML1) (Zhang et al., 2012). Apo-Tf is then released from TfR1 and recycled back to the cell membrane to repeat another cycle (Pantopoulos et al., 2012). Thereafter, the newly acquired iron enters into the redox-active “labile iron pool” in the cytosol (Gkouvatsos et al., 2012; Pantopoulos et al., 2012). In addition, cellular iron balance is post-transcriptionally regulated by two iron regulatory proteins, namely, IRP1 and IRP2. Under Fe-deficient conditions, IRP1 and IRP2 specifically bind to iron-responsive elements in 3′- or 5′-UTR of the mRNA transcripts of TfR1, ferritin H chain (Fth1), ferritin L chain, or DMT1; as a result, these regulatory proteins are protected from degradation or their translation is inhibited (Anderson and Vulpe, 2009; Dunn et al., 2007; Kaplan and Kaplan, 2009; Muckenthaler et al., 2008).

## Iron-requiring proteins and dna replication/repair

Numerous proteins involved in DNA replication and repair require iron as a cofactor. These proteins include the three DNA polymerases (Pol α, Pol δ and Pol ε), the DNA helicases (Rad3/XPD, Dna2, RTEL1, FANCJ and ChlR1), and DNA primase regulator subunit Pri2 (PRIM2 in mammals). Moreover, the eukaryotic RNR small subunit requires iron to form a diferric-tyrosyl radical cofactor (Fe_2-_^III^Y∙) to initiate nucleotide reduction.

### DNA polymerases/primases and DNA replication

Although the eukaryotes contain diverse genomic sizes, the bulk of DNA synthesis is performed via three conserved polymerases: Pol α, Pol δ and Pol ε (Miyabe et al., 2011). Pol α tightly associates with DNA primases to initiate the synthesis of short RNA primers that are further utilized by Pol δ and Pol ε to synthesize the lagging and leading strands, respectively (Schumacher et al., 2000; Wang et al., 2004). Generally, eukaryotic DNA primases are heterodimeric enzymes with a small (PriS) and a large (PriL) subunit, in which the PriL subunit contains a conserved Fe-S domain necessary to initiate DNA replication (Kilkenny et al., 2013; Prakash and Prakash, 2002; Sauguet et al., 2010). Eukaryotes also possess Pol ζ, a B-family polymerase with lower fidelity than other polymerases; Pol ζ specifically functions in the extension step of translesion DNA synthesis (Acharya et al., 2006). All of these DNA polymerases and primases require a Fe-S cluster for the formation of active holoproteins, implying the importance of iron in maintaining genome integrity (Netz et al., 2012). Interestingly, the functions of these nuclear DNA polymerases depend on the cytosolic and the mitochondrial Fe-S protein biogenesis machineries, presumably because they act as sulfur donors for the Fe-S cluster in DNA polymerases (Rouault, 2012).

### DNA helicases, DNA replication and repair

DNA helicase and helicase-nuclease enzymes, including XPD, Rad3, FancJ, ChlR1, RTEL1 and Dna2, preserve genome stability and are genetically associated with diseases characterized by DNA repair defects (Rudolf et al., 2006; Wu et al., 2009; Wu et al., 2012). Each of these proteins contains a conserved Fe-S cluster near the N-terminus, which is essential for helicase activities (Wu et al., 2012). XPD is classified as a SF2- DNA helicase and plays an important role in nucleotide excision repair (NER). FANCJ can catalytically unwind duplex DNA and G-quadruplex structures in an ATP hydrolysis-dependent manner (Wu et al., 2012). Clinically, patients with trichothiodystrophy (TTD) and Fanconi anemia completely lose helicase activities because of the relevant mutations in the Fe-S clusters of XPD and FancJ proteins, respectively (Coin et al., 2007; Fregoso et al., 2007). Moreover, site-directed mutagenesis of four conserved cysteines in Fe-S cluster of yeast XPD (Rad3) leads to defects in excision repair of UV photoproducts (Wu et al., 2009). The Fe-S cluster in the Rad3 helicase is necessary to induce the coupling of ATP hydrolysis with DNA translocation and to target helicase in the ss DNA-ds DNA junction (Pugh et al., 2008). ChlR1 is a DEAH/DEAD box-containing DNA helicase belonging to the FANCJ-like DNA helicase family. The mammalian and yeast ChlR1 proteins facilitate the establishment of sister chromatid cohesion and the maintenance of genomic stability. In *Caenorhabditis elegans*, CHL-1 is essential for normal development, fertility and chromosomal stability (Laha et al., 2011; Parish et al., 2006); RTEL specifically interacts with the shelterin complex and involves in telomere maintenance in mammals (Wu et al., 2012). These DNA helicases generally function together with other transcription factor II H (TFIIH) complex members such as XPG, XPB, p62, p52, p44, p34, p8/TTDA, Cdk7, cyclin H and MAT1 in human, and Rad3, Rad25, TFB1, SSL1, p55 and p38 in yeast (Sung et al., 1996).

The SF1 Dna2 helicase-nuclease, a protein implicated in double-strand break (DSB) end resection and Okazaki fragment processing, also contains a Fe-S cluster (Wu et al., 2012). Mutations in the Fe-S domain of Dna2 affect the ability of protein complexes to bind broken DNA, thereby impairing DNA replication; this result indicates the essential function of Fe-S in this process (Wu et al., 2012). Moreover, several other DNA helicases, such as DinG (*E. coli*), AddAB (*E. coli*), and DOG-1 (*C. elegans*), are implicated in DNA replication and repair (Wu et al., 2012).

### Other iron-sulfur cluster proteins and DNA replication

The maturation of mitochondrial Fe-S proteins is carried out via the iron-sulfur cluster (ISC) assembly machinery, whereas cytoplasmic and nuclear Fe-S protein biogenesises depend on both the ISC and CIA (cytosolic iron-sulfur cluster assembly) machineries (Lill et al., 2012; Sipos et al., 2002). The components and biogenesis mechanisms of CIA pathway exhibit high degree of conservation in mammals and yeast. Yeast cells assemble a transiently bond [4Fe-4S] cluster on the Nbp35-Cfd1 scaffold (NUBP1-NUBP2 in mammals). This synthesis reaction further requires the electron transfer chain from Dre2 (CIAPIN1 in mammals) and its binding partner, the diflavin NADPH oxidoreductase Tah18 (NDOR1 in mammals). Thereafter, the transiently bound [4Fe-4S] cluster of Cfd1-Nbp35 is transferred to apoproteins, including apo-Cia1 (CIAO1 in mammals), apo-Nar1 (NARFL in mammals), apo-Cia2 and apo-MMS19, to form their corresponding holoproteins (Couturier et al., 2013; Zhang et al., 2008). The yeast MMS19 is necessary to transfer Fe-S clusters to target proteins and is also identified to affect DNA repair, chromosome segregation and heterochromatin silencing (Stehling et al., 2012). More importantly, both the human and yeast MMS19 proteins interact with numerous Fe-S proteins, including Pol δ, DNA primase, Dna2, XPD, RTEL1 and FANCJ (Gari et al., 2012). The stabilities of these Fe-S proteins are severely affected in the absence of MMS19 (Gari et al., 2012). Consistent with its essential role in DNA replication and repair, MMS19 deficiency in yeast or human cells exhibits increased sensitivity to hydroxyurea and S phase defect during cell cycle. Moreover, MMS19 can form a complex with two other CIA machinery proteins, namely, Cia1 and Cia2, suggesting that these CIA proteins are possibly involved in DNA replication and repair (Stehling et al., 2013).

The iron regulatory protein IRP1 possesses a [4Fe-4S] cluster. The inhibition of the IRP1 aconitase activity in L5178Y mouse lymphoma cells can increase “labile iron pool” levels. The increased iron burden in LIP leads to exacerbated hydrogen peroxide-induced genotoxicity in L5178Y cells (Lipinski et al., 2005). The stable IRP1 knockdown by shRNA interference in HL60 radiosensitive cells causes radioresistance to linear energy transfer gamma rays, but a more rapid DNA DSB repair. The mechanism of radioresistance is possibly related to the attenuated free radical-induced cell death (Haro et al., 2012).

### RNR, iron, DNA replication and DNA repair

RNRs are enzymes that use radical chemistry to reduce ribonucleotides to synthesize deoxyribonucleotides (dNTPs), thereby generating the necessary precursors of DNA replication and repair (Zhang et al., 2014a, b). Imbalanced dNTP pools usually lead to increased DNA mutations, DNA breaks and cell death by enhancing misincorporation and by inhibiting the proofreading function of DNA polymerases (Kumar et al., 2010; Zhang et al., 2014a, b). Eukaryotic RNRs comprise the large subunits (α or R1) and small subunits (β or R2). Similar to Fe-S proteins, the RNR small subunits also require iron to sustain a diferric tyrosyl radical (Fe_2-_^III^Y∙) cofactor. Cells depleted of Grx3/4 exhibit deficiencies in RNR nucleotide reduction activity, but the mechanism remains to be elucidated (Zhang et al., 2008; Li et al., 2009; Netz et al., 2010, Ueta et al., 2012). Interestingly, the recent studies have shown that the depletion of Dre2 affects both *RNR* gene transcriptions and mRNA turnover by activating Aft1/Aft2-controlled iron regulon (Zhang et al., 2014a, b). The RNR subunit protein levels are tightly regulated by the DNA damage checkpoint. For instance, the mammalian small subunit RRM2 is degraded via an ubiquitin-mediated mechanism when cells complete DNA replication and/or repair. This process is mediated through two E3 ubiquitin ligase complexes, namely, the Skp1/Cullin/F-box (SCF) and the anaphase-promoting complex (APC) (Chabes et al., 2003b; D’Angiolella et al., 2012). In response to DNA damage, mammalian cells increase the transcription of *p53R2* (*RRM2B*), which further forms an active RNR holoenzyme with RRM1 to facilitate DNA repair by activating the ATM/ATR-CHK checkpoint pathway (Harper and Elledge, 2007; Nakano et al., 2000). Similarly, the expressions of yeast *RNR* genes are induced via the activation of the Mec1-Rad53-Dun1 damage checkpoint kinase cascade, particularly *RNR3* (Zhang et al., 2014a, b).

### Hemoproteins and DNA stability

Heme commonly serves as the prosthetic group for hemoproteins, such as hemoglobin, myoglobin, cytochromes and nitric oxide synthase. These hemoproteins are involved in oxygen transport, oxidative catalysis and electron transport (Rae and Goff, 1998; Brown et al., 2004; Pamplona et al., 2007; Girvan and Munro, 2013). In addition, heme is important for systemic iron homeostasis in mammals, as it is present in many normal dietary sources (Pamplona et al., 2007). A number of heme transporters (FLVCR, ABCG2/BCRP, ABCB6, ABCB7 and ABCB10, PCFT/HCP1, HRG-1 and HRG-4) and heme-binding transcription factors (Bach1, NPAS2 and Rev-erb) are reportedly involved in heme metabolism and regulation (Severance and Hamza, 2009; Yang et al., 2008). Many hemes are enzymatically degraded by their degradation systems, such as heme oxygenases (HO, including HO-1, 2, and 3) and microsomal cytochrome P450 reductase. A considerable amount of hydrogen peroxide (H_2_O_2_) is produced during heme degradation, which may cause cellular toxicity and DNA damage (Quincozes-Santos et al., 2013; Wagener et al., 2003).

The disruption of hemoproteins, such as cytochromes *b*_5_ and nitric oxide synthase, possibly increases ROS production. Cytochromes *b*_5_ is a membrane bound hemoprotein and generally serves as an electron carrier in several oxidative reactions of reductases, such as NADH-cytochrome *b5* reductase (Reid et al., 2013; Schenkman and Jansson, 2003; (Vergeres and Waskell, 1995), NADPH-cytochrome P450 reductase (Gan et al., 2009; Pyrih et al., 2014), and fatty acid desaturases involved in lipid and cholesterol biosynthesis (Reddy et al., 1977; Keyes and Cinti, 1980; Larade et al., 2008). The yeast Irc21, which shares similar heme-binding sites with Cyb5, reportedly functions in chromatin remodeling and the increase of DNA damage foci (Alvaro et al., 2007). This result indicates that Irc21 may be involved in DNA replication process, and the assumption is supported by the genetic interactions of Irc21 with several DNA damage- and repair-related proteins, such as Pri2, Pol12, Dia2, Rad17, MMS22 and CDC13 in *Saccharomyces* genome database (SGD). Moreover, cytochrome *c* is also a small hemoprotein involved in apoptosis and cell death (Jiang et al., 2004).

## Anemia and dna stability

Anemia occurs as a result of numerous underlying causes and can be classified into different types based on the morphologies of red blood cells, discernible clinical spectra and etiologic mechanisms (Shah and Agarwal, 2013). As the most common form of anemia, iron deficiency anemia increases nuclear DNA damage in adults, as demonstrated by an increased DNA damage in anemic subjects (Aslan et al., 2006). Conversely, the results of iron nutritional deficiency in rats do not affect DNA stability or lipid peroxidation (Diaz-Castro et al., 2008). Studies have also indicated that dietary iron-deficient anemia induces various metabolic changes and even apoptosis in rat liver (Kamei et al., 2010). Moreover, fanconi anemia, a genetic disorder, is caused by defects in a cluster of proteins responsible for DNA repair. Studies have shown that eight of these proteins (FANCA, -B, -C, -E, -F, -G, -L and -M) assemble to form a core protein complex in the nucleus. Their assemblies are activated by replicative stresses, particularly DNA cross-linking agents (mitomycin C or cisplatin) and ROS (Deans and West, 2009). The deficiency of several ribosomal proteins (RP) can cause diamond blackfan anemia (DBA), which is a genetic syndrome characterized by red blood cell aplasia. RP-deficient zebrafish and human hematopoietic activate the ATR/ATM/CHK1/2/p53 pathway (Danilova et al., 2014).

## Iron-requiring proteins and cell cycle control

Iron is a major regulator of cell cycle by inhibiting the formation or activities of the cyclin and cyclin-dependent kinase complexes. Intracellular iron disruption by chelators causes cell cycle arrest, particularly in G_1_ and S phases (Fu et al., 2007; Siriwardana and Seligman, 2013).

### Cyclin, iron and cell cycle

The yeast cell cycle is mainly regulated by CDK (Cdc28), together with two other cyclin families: Cln1–3 and Clb1–6 (Nasmyth, 1993; Mendenhall and Hodge, 1998). Cdc28 associates with different cyclin proteins to govern cell cycle issues. For instance, Cln1/Cdc28 and Cln2/Cdc28 function in budding generation and spindle pole body duplication; Cln3/Cdc28 controls the size of newly formed cells (Chen et al., 2000). Clb1/Cdc28 and Clb2/Cdc28 are involved in mitosis; Clb3/Cdc28 and Clb4/Cdc28 are implicated in DNA replication and spindle formation, Clb5/Cdc28 and Clb6/Cdc28 are required for DNA replication (Mendenhall and Hodge, 1998; Ofir and Kornitzer, 2010; Vohradsky, 2012).

Constant *AFT1* expression results in an increased iron uptake, thereby leading to cell cycle arrest at the start of G_1_ regulatory point (Philpott et al., 1998). The expression of the G_1_-specific cyclins (Cln1 and Cln2) is decreased when yeast cells are exposed to iron rich conditions, which may account for the arrest (Philpott et al., 1998; Yu et al., 2007).

In human, cyclins and CDK also control cell cycle progression. Intracellular iron depletion by some chelators causes allosteric inhibition of cyclin-A, cyclin-E, Cdc2 and Cdk2, resulting in cell cycle arrest in G_1_ and S phases (Renton and Jeitner, 1996; Yu et al., 2000). In some cases, intracellular iron depletion reduces cyclin-D and Cdk4 protein levels and alters retinoblastoma protein phosphorylation (Yu et al., 2000). This G_1_/S arrest further indicates the essential roles of iron in cell cycle progression, growth and division. Under some iron deficient conditions, a G_2_/M arrest has also been detected (Renton and Jeitner, 1996). Furthermore, several iron chelators evidently exhibit strong anticancer activities by inducing cell cycle arrest and apoptosis (Rao, 2013). However, involved mechanisms are barely known; as such, the relationships between iron chelators and structural activities should be understood.

### Iron-sulfur cluster proteins and cell cycle

In yeast, the expressions of iron transporter genes *FET3*/*FTR1* are tightly regulated by cell cycle and reach the peaks during M and M/G_1_ phases (Spellman et al., 1998). Two tah18 temperature-sensitive (ts) mutants, namely, tah18-5I5 and tah18-5H8, exhibit a prolonged S phase and a delay at the G_1_/S boundary, respectively (Zhang et al., 2014a, b). *MMS19* gene silencing in human cells leads to genotoxic stress sensitivity (Stehling et al., 2012) and G_1_ phase arrest in cell cycle under limited nucleotide pool conditions, suggesting that DNA replication is impaired in these cells (Gari et al., 2012).

In human, the upregulation of CIAPIN1 results in significant inhibition of the CCRCC-derived cell growth *in vitro* and *in vivo* with G_1_ cell cycle arrest (He et al., 2009). CIAPIN1-induced growth suppression reduces protein levels of cyclin D1, cyclin E, Cdk2, Cdk4, p-Rb and VEGF (He et al., 2009). Moreover, Grx3 is critical for cell cycle progression during embryogenesis in mouse. Cells depleted of Grx3 undergoes normal DNA replication during the S phase but exhibit impaired growth and cell cycle progression at the G_2_/M phase (Cheng et al., 2011).

### Iron-requiring proteins and cell cycle

In budding yeast, information on the hemoproteins involved in cell cycle is limited. However, heme and hemoproteins have been implicated in controlling the expressions of cell cycle regulators and cell growth in mammals. Heme synthesis inhibition causes cell cycle arrest in S phase, upregulates p53 and CDK inhibitor p21 protein levels, and downregulates Cdk4, Cdc2 and cyclin D2 protein levels (Ye and Zhang, 2004). The overexpression of heme oxygenase-1 (HO-1) in human pulmonary epithelial cells results in cell growth arrest during G_0_/G_1_ phase and increased resistance to hyperoxia (Lee et al., 1996).

### RNR and cell cycle

RNR expression and activity are strictly regulated during cell cycle to generate and maintain proper dNTP pools that ensure the fidelity of DNA synthesis and repair (Tanaka et al., 2000; Sanvisens et al., 2011). Generally, the dNTP pools increase by 5- to 10-fold when cells transit from G_0_/G_1_ phase to S phase; this transition is mainly achieved by increased RNR levels during the exponential phase in bacteria and the S phase in eukaryotes (Cendra Mdel et al., 2013; Zhang et al., 2014a, b). In mammalian cells, the transcriptions of *RRM1* and *RRM2* are dependent on cell cycle with low or undetectable transcription levels in G_0_/G_1_ phase but maximal levels during S phase (Bjorklund et al., 1990; Chabes et al., 2004). In plants, the expressions of *R1* and *R2* are also S phase specific and dependent on the E2F-like motifs in their promoters (Chaboute et al., 2002). In budding yeast, the transcriptions of RNR genes (*RNR1*, *RNR2* and *RNR4*) peak at the beginning of the S phase by regulating the transcription factor pairs Mbp1/Swi6 and Swi4/Swi6, which can bind to the Mlul cell cycle box (Sanvisens et al., 2013). However, no cell cycle regulation has been observed in *RNR3* transcription (Lee and Elledge, 2006; Sanvisens et al., 2013).

## Summary

Although results have indicated that iron-requiring proteins are implicated in DNA replication, repair and cell cycle control, limited information is available regarding their functional mechanisms. Several iron-requiring proteins, such as DNA polymerases/primases, DNA helicases, and RNRs, directly participate in DNA replication and repair. Biochemical and structural studies have suggested that Fe-S domains in these enzymes serve a structural rather than a redox-active function by possibly stabilizing local domain conformation that may mediate protein-protein or protein-nucleic acid interactions. Disruption of some iron-requiring proteins, particularly hemoproteins, associates with the generation of ROS, which results in DNA damage. Mutations of iron-requiring proteins are associated with diseases characterized by DNA repair defects and/or a poor response to replication stress in mammals. Thus, a detailed understanding of the mechanisms of Fe-requiring protein functions may provide insights into the related mutagenic diseases.
